# Atrial fibrillation following major noncardiac thoracic surgery: significance and impact on morbidity

**DOI:** 10.1186/cc11080

**Published:** 2012-03-20

**Authors:** H Michalopoulou, PS Stamatis, M Michaloliakou, N Baltagiannis, D Stamatis

**Affiliations:** 1 'Metaxa' Hospital, Athens, Greece

## Introduction

Atrial fibrillation (AF) is a common complication after noncardiac thoracic surgery. Its impact on overall mortality has not yet been fully assessed and few data are available on the effects of the noncardiac post-thoracotomy AF on clinical outcomes.

## Methods

From July 2006 to July 2011, 226 consecutive patients undergoing lung resection for lung cancer were studied retrospectively. Preoperative data and serial electrocardiograms were evaluated. Hypertension, dyslipidaemia, diabetes mellitus, smoking and advanced age (>75 years) were considered as risk factors. Patients (*n *= 97) who had structural heart disease or ≥2 risk factors were considered a high-risk group whereas those with <2 risk factors constituted the low-risk group.

## Results

Thirty-two patients (14.16%) experienced new-onset post-operative AF. The high-risk group had a 58% incidence of AF compared with 23% in the low-risk group (*P *< 0.001). Moreover, following β-blocker administration, more of the high-risk group required antiarrhythmic treatment with amiodarone than did the low-risk group (67% vs. 35% respectively, *P *= 0.02). Patients who developed AF had a significantly longer hospital stay (*P *< 0.01). The 30-day mortality rate was significantly higher in the high-risk group (11% vs. 2%; *P *= 0.03) but AF was not an independent risk factor for death. In the multivariate analysis, major resection (pneumonectomy) and advanced age were identified as independent risk factors for the development of postoperative AF (*P *= 0.004 and *P *= 0.008 respectively).

## Conclusion

Atrial fibrillation occurrence after lung resection does not independently affect the short-term mortality but is associated with a prolonged length of hospital stay.

